# Emerging biologic augmentation strategies for meniscal repair: a systematic review

**DOI:** 10.1186/s12891-024-07644-2

**Published:** 2024-07-13

**Authors:** David Mazy, Jessica Wang, Philippe Dodin, Daisy Lu, Florina Moldovan, Marie-Lyne Nault

**Affiliations:** 1 CHU Sainte-Justine, Montréal, 7905-3175, Côte Ste-Catherine, QC H3T 1C5 Canada; 2 Faculty of Medicine, Université de Montréal, 2900 Boul. Edouard-Montpetit, Montreal, QC H3T 1J4 Canada; 3 CHU Sainte-Justine Azrieli Research Center, Montréal, 7905-3175 Côte Ste-Catherine, H3T 1J4 QC Canada; 4 Department of Orthopedic Surgery, CIUSSS Hôpital du Sacré-Cœur de Montréal (HSCM), 5400, Boul. Gouin Ouest, Montreal, QC H4J 1C5 Canada

**Keywords:** Meniscal repair, Biologic augmentation, Growth factors, Cell therapy

## Abstract

**Background:**

Meniscal repair should be the gold standard. However, the meniscus is poorly vascularized and even an excellent meniscus repair may not heal. Therefore, numerous studies and systematic reviews have been carried out on platelet-rich plasma (PRP), mesenchymal stem cells (MSCs) and fibrin clots for meniscal augmentation, but the results remain controversial. This systematic review aimed to identify other emerging strategies for meniscal repair augmentation and to assess whether there are different avenues to explore in this field.

**Methods:**

A systematic literature review was conducted in August 2022. PubMed, Ovid MEDLINE(R) all, Ovid All EBM Reviews, Ovid Embase and ISI Web of Science databases were searched. In Vivo animal and human studies concerning the biological augmentation of meniscal lesions by factors other than PRP, MSCs or fibrin clots were included. Cartilage-only studies, previous systematic reviews and expert opinions were excluded. All data were analyzed by two independent reviewers.

**Results:**

Of 8965 studies only nineteen studies covering 12 different factors met the inclusion criteria. Eight studies investigated the use of growth factors for meniscal biologic augmentation, such as vascular endothelial growth factor or bone morphogenic protein 7. Five studies reported on cell therapy and six studies focused on other factors such as hyaluronic acid, simvastatin or atelocollagen. Most studies (*n* = 18) were performed on animal models with gross observation and histological evaluation as outcomes. Polymerase chain reaction and immunohistochemistry were also common. Biomechanical testing was the object of only two studies.

**Conclusions:**

Although several augmentation strategies have been attempted, none has yielded conclusive results, testifying to a lack of understanding with regard to meniscal healing. More research is needed to better understand the pathways that regulate meniscus repair and how to act positively on them.

**Level of evidence:**

Systematic review of case–control and animal laboratory studies.

## Background

The meniscus plays an essential role in load distribution, stabilization, proprioception, lubrication, and nutrition for the knee joint. It is mainly composed of water, type 1 collagen, proteoglycans and a few rare fibrochondrocytes and stem cells. In adults, the meniscus receives a limited, peripheral blood supply with poor healing potential [1]. Unfortunately, meniscus injuries are extremely common, whether acute or degenerative [2]. Indeed, arthroscopy for meniscal injury is the most frequently performed orthopedic procedure [3, 4]. The current dogma in meniscus surgery is "save the meniscus" and although meniscectomy rates are decreasing, this procedure is still widely performed [5, 6]. The benefits of meniscectomy include not only excellent early post-operative outcomes, but also being easy to perform, fast, and cheaper than meniscal repair [7]. Unfortunately, this surgery leads to early-onset osteoarthritis (OA), both radiologically and clinically [8, 9]. Meniscal repairs are therefore increasingly performed with success rates between 60% and 90% with the appropriate indications and depending on the type of tear [10, 11]. However, some tears cannot be sutured or are located in avascular areas, which is detrimental to their healing potential.

Moreover, even with a well-performed suture, the rate of secondary meniscectomies remains high, ranging from 15% to 24%, indicating that the meniscal tissue did not heal [10, 11]. Therefore, the scientific community turned to biological augmentation techniques to improve healing rates [12]. Several meta-analyses focusing on platelet-rich plasma (PRP), mesenchymal stem cells (MSCs), and fibrin clots exist, as well as a few comparative studies [13–15]. Unfortunately, the results do not support one type of augmentation strategy over the two others in a clear-cut fashion. The aim of this systematic review was to move beyond these three typically studied factors and identify emerging factors that should be the subject of further study for the biological augmentation of meniscal tears.

## Methods

### Search Strategy

The Preferred Reporting Items for Systematic Reviews and Meta-Analyses (PRISMA) were used to carry out a systematic review of emerging factors (excluding PRP, MSCs and fibrin clot) used in research for meniscal augmentation. Searches of the PubMed, Ovid MEDLINE(R) all, Ovid All EBM Reviews, Ovid Embase, and ISI Web of Science databases were completed in August 2022 by a librarian from our institution specializing in literature searches. The basic keywords used were: (1) “meniscus” or “meniscal” or “medial meniscus” or “lateral meniscus” or “meniscus injury” or “meniscal tear”; (2) “repair” or “surgery” or “augmentation” and (3) “biological augmentation” or “growth factor” or “cell therapy”.

### Study selection and data extraction

The inclusion criteria for screening were: in vivo studies on strategies that may optimize meniscal healing, investigating humans or animals, in English, and from 1980 onwards. The exclusion criteria were: studies using PRP, MSCs or fibrin clots as meniscal repair augmentation strategies, in vitro studies, cadaveric studies, previous systematic review or meta-analyses, recommendations and guidelines, studies only on osteoarthritis, veterinary studies, ophthalmology and maxillofacial studies (meniscal tear is also a pathology in ophthalmology). In each of these studies, the authors, title, study design, animal or human, type of animal, number of specimens studied, type of meniscal tears performed, form of factor studied (liquid, solid or combined with a matrix), outcomes, advantages and disadvantages of each factor were extracted. Two independent researchers, one of whom is an orthopedic surgeon and the second a medical student, screened the studies. A senior author was available to resolve any disagreements. The first step was to screen the titles of the articles, the second step was to screen the abstracts of the selected articles and the third step was to complete a review of the full manuscript.

## Results

**Fig. 1:**
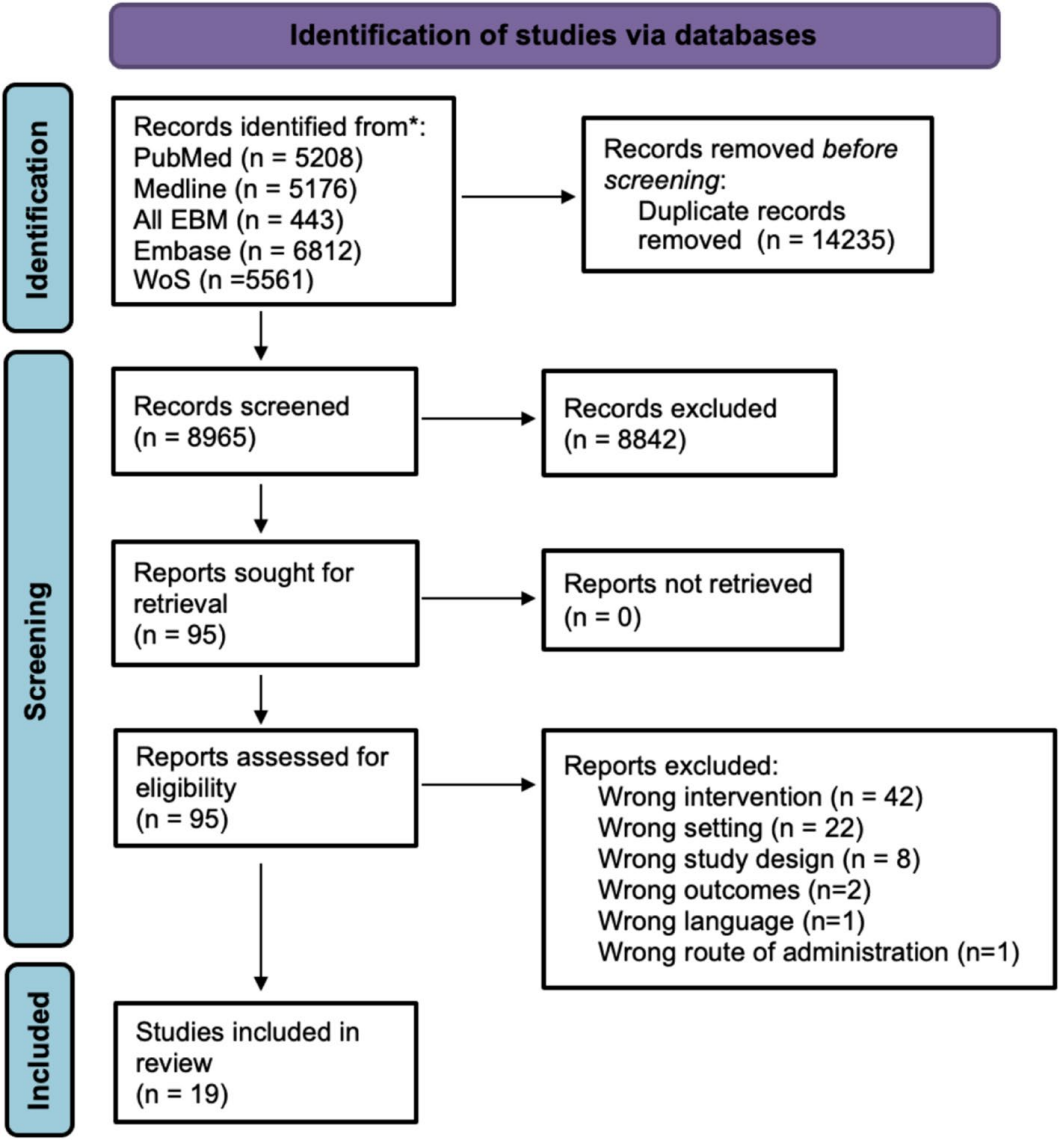
PRISMA flow diagram

**Fig. 2:**
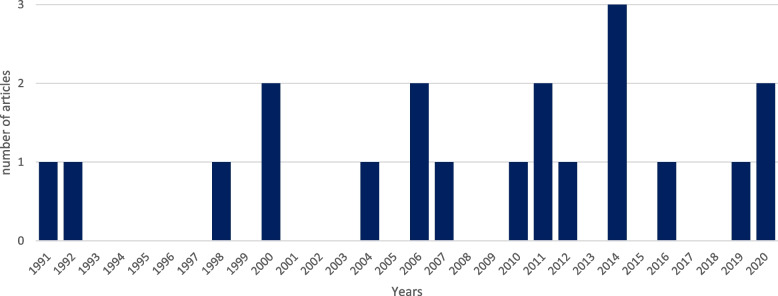
Years of publication of the 19 extracted studies

A total of 23,200 citations were retrieved from the 5 databases. Duplicates were removed in EndNote by the librarian, leaving 8965 records to screen, which were imported into Covidence. After screening the titles and the abstracts of the 8965 studies, only 95 were relevant for the full-text screening, excluding 8842 studies. Most of the studies were excluded because they were about osteoarthritis in a general sense, focused on augmentation of cartilage repair instead of meniscus repair or on meniscal transplantation, etc. Following the full-text screening of the 95 remaining articles, a total of 19 studies were included for data extraction. These 76 articles were excluded because of: wrong intervention (*n* = 42), wrong setting (*n* = 22), wrong study design (*n* = 8), wrong outcomes (*n* = 2), wrong language (*n* = 1), and wrong route of administration (*n* = 1) (Fig. 1). Of the 19 studies included, only one was on humans, leaving 18 performed on animals. Publication years ranged from 1991 to 2020, with 53% (10/19) published from 2010 onwards (Fig. 2). The human study was a retrospective case–control level III study, whereas all the animal models were basic science studies. In the human study, 47 medial menisci from 47 participants were included [16]. In the animal studies, there were 598 specimens including 44 sheep, 15 dogs, 415 rabbits, 56 rats, 16 pigs, 12 goats, 1 mouse and 39 pigs, for a total of 746 menisci. In the animal models, the most common type of menisci studied was the medial menisci (*n* = 436), followed by uncategorized menisci (*n* = 189) and the lateral menisci (*n* = 121).

**Fig. 3:**
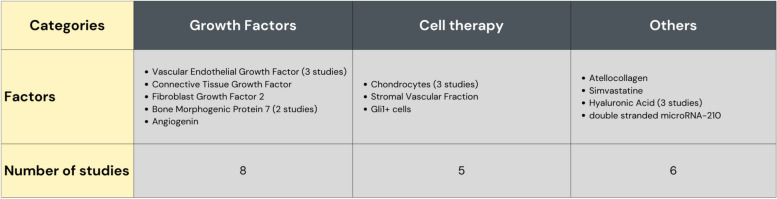
Distribution of factors by categories

The augmentation technique for meniscus repair studied in humans was atelocollagen. The augmentation techniques tested on animals included three main categories: growth factors, cell therapy and “others”. With regard to growth factors, vascular endothelial growth factor (VEGF) was the subject of three studies, bone morphogenic protein 7 (BMP-7) of two studies, while connective tissue growth factor (CTGF), fibroblast growth factor 2 (FGF-2) and angiogenin were the subject of one study each. In terms of cell therapy, chondrocyte implantation was the most studied strategy, with three studies, while stromal vascular fraction (SVF) and Gli1 + cell therapy were the subject of one study each. In the category "other", we found three studies on the use of hyaluronic acid, one on simvastatin and one on microRNA for meniscal augmentation (Fig. 3).

In terms of outcomes, of the 18 animal studies, 94% performed histological analysis after sacrifice, 78% gross observation (macroscopic aspect), 39% immunohistochemical (IHC) analysis and 22% polymerase chain reaction (PCR). Only one animal study involved a magnetic resonance investigation [17]. The outcomes for the human study were clinical (Lysholm score and International Knee Documentation Committee) and magnetic resonance [16].

## Discussion

This study brings to the forefront how difficult it is to identify an emerging factor of interest for meniscal augmentation. In fact, although many factors have been studied, none have provided sufficient evidence to hope for a sustainable clinical transposition. The heterogeneity of study designs and study groups, as well as the diversity of factors studied, make it impossible to compare them effectively and to perform a meta-analysis. In our opinion, this wide variety of factors studied reflects the significant knowledge gap that scientists are faced with when it comes to meniscus tissue and its metabolism [18]. More generally, the paucity of articles on emerging technologies reflects a lack of interest in factors other than PRP, MSCs or fibrin clot. On the other hand, the number of articles published over the years shows a growing interest over the last decade, perhaps due to the absence of formal proof of efficacy for the three factors most frequently used in clinical trials [13].

The following is a description and analysis of the various studies found in this systematic review. They are classified according to the three categories described in the results and according to the factor studied.

### Growth factors

#### Vascular endothelial growth factor (VEGF)

VEGF induces angiogenesis through its ability to promote endothelial cell division [19]. There were three studies on VEGF for meniscal repair augmentation. As early as 1992, Hashimoto et al. carried out a study on 15 dogs.

They created 2 mm diameter circular defects in the avascular zone and compared three groups (*n* = 1) at different sacrifice times (1, 2, 6, 12, and 24 weeks). Defect filling with fibrocartilaginous tissue was significantly better, both macro- and histologically, when VEGF was applied within fibrin sealant. However, this tissue transformed into hyaline cartilage-like tissue by 12 weeks, rather than meniscus-like tissue. Another use for VEGF was to incorporate it into a suture coating [20]. Petersen et al. compared three groups (*n *= 6 sheep), all with longitudinal tears in avascular zones [21]. One group was treated with Ethibond sutures, the second with poly(d,l-lactide)-coated Ethibond (PDLLA coated suture) and the third with VEGF incorporated into the PDLLA coating. After sacrifice at 6 weeks, no scarring was observed macroscopically or histologically in the VEGF group, whereas scar tissue could be observed in the other two groups. The latest VEGF study used the same suture-coated methodology, on 18 sheep, but with a sacrifice at 8 weeks [22]. In addition to macroscopic evaluation, immunohistochemistry and PCR were performed, but unfortunately failed to show any improvement in meniscal healing or angiogenesis compared with control groups. Moreover, they were able to demonstrate almost complete VEGF clearance at 9 days. Although VEGF is theoretically a very interesting growth factor for poorly vascularized tissue, there is currently no evidence to suggest that it is optimally suited to meniscal augmentation.

#### Connective tissue growth factor (CTGF)

CTGF is a growth factor that promotes fibroblast adhesion, migration and survival, as well as chondrocyte proliferation and differentiation [23]. CTGF also has a role, in vitro, in neoangiogenesis [24]. Theoretically, these properties make it interesting for meniscus repair augmentation. He et al. carried out an in vivo study on rabbits [24, 25], performing longitudinal tears in a red-white zone (between vascular and avascular zones). They then compared three groups (*n* = 5): simple sutures, sutures with fibrin glue and sutures with CTGF incorporated in fibrin glue. Sacrifices were performed at 1, 4 and 10 weeks. The PCR analysis found that the CTGF-treated group showed greater expression of type 1 and type 2 collagen, as well as greater VEGF expression at 4 and 10 weeks post-operatively, compared with the control groups. Fluorescence-immunohistochemical imaging confirmed these findings. These results make CTGF a factor of interest, despite the need for more extensive studies, particularly in avascular areas, but also about the biomechanical properties of the scar tissue.

#### Fibroblast growth factor (FGF-2)

FGF-2 is known to promote fibroblast proliferation and angiogenesis [26]. Narita et al. studied this factor for the augmentation of horizontal tears in rabbit menisci [26]. Partial horizontal tears starting from the menisco-capsular junction, and thus crossing the red-red zone, were produced. When FGF-2 was combined with a gelatine hydrogel matrix (secured by a suture), it appeared to produce a better histological healing score and inhibited the death of meniscal cells for up to 4 weeks compared to control groups. Although very interesting, the type and zone where the tear was performed, as well as the absence of biomechanical tests mean that further studies on this factor are required.

#### Bone morphogenic protein 7 (BMP-7)

BMP-7 is a growth factor already used in orthopedics for its osteogenic and embryogenic cascade induction capabilities in the treatment of recalcitrant long bone nonunion [27]. Two articles studied BMP-7 for meniscal augmentation in an in vivo animal setting. In Forriol et al., 4 mm punch holes were made in the medial menisci (*n* = 16) of 8 sheep [28]. The study group was treated with BMP-7 in a matrix of bovine collagen and the control group with this same matrix alone. In the study group, the presence of cells with fibrous tissue composed mainly of type 2 collagen was much greater than in the control group on histological sections. In addition, BMP-7 clearance was estimated at between 1 and 8 days.

In Zellner et al. 2 mm punch holes were made in the menisci of 12 white rabbits. These were divided into a study group, treated with an injection of BMP-7, and a control group without injection [29]. The experimental treatment consisted of BMP-7 injected into a cell-free hyaluronan collagen composite matrix. They were unable to demonstrate any advantage of using BMP-7 either macroscopically, histologically or immunohistochemically. Although BMP-7 has interesting properties, its ability to develop a matrix composed of collagen type 2 makes it an unlikely candidate to repair the human meniscus which essentially consists of collagen type 1. Moreover, its rapid clearance means that it needs to be combined with a matrix to slow down its release.

#### Angiogenin

Back in 1991, King et al. considered inducing neovascularization to promote healing of the avascular zone of the meniscus [30, 31]. To achieve this, they implanted dry cellulose discs containing angiogenin into pockets created in longitudinal tears on rabbit external menisci. The treated group showed better local neovascularization, but not better healing of the meniscus tissue itself. Angiogenin, although different from VEGF, is also considered a growth factor and a strong stimulator of angiogenesis [32]. Despite its potential, this animal study failed to recommend angiogenin as a meniscal augmentation factor.

### Cell therapy

#### Chondrocytes

Three articles described the use of chondrocytes (auto or allogenic) to enhance meniscal repair [32–35]. Peretti et al. carried out a study on 16 pigs with a longitudinal tear in the avascular portion of the left medial meniscus [32, 33]. Four different groups (*n* = 4) were set up: an untreated group, a sutured group, a group treated with a simple scaffold and a group treated with a scaffold seeded with autologous chondrocytes. The scaffold was a slice of allogenic meniscus secured to the lesion by 2 sutures. Pigs were sacrificed at 9 weeks and outcomes included gross observation and histology. The internal avascular third of the meniscus, or W-W zone, showed no increased vascularization. However, meniscal healing was macroscopically and histologically greater in the cell therapy group. Weinand et al. conducted two studies on the use of chondrocytes for meniscal augmentation in pigs [33, 34]. The chondrocytes had two origins: allogenic, from articular, auricular and costal cartilage, or autologous and derived from the same areas. For delivery, chondrocytes were seeded in a Vicryl mesh scaffold. Sacrifice was at 12 weeks, and macroscopic and histological analyses were performed. The results favored the chondrocyte-treated groups but showed no difference between allogenic and autogenic strategies. Although interesting, autologous chondrocyte transplantation has some drawbacks, namely the need for two procedures and donor site comorbidity [35]. In addition, the scar tissue composition is more cartilage-like than meniscus-like and could be better characterized by PCR.

#### Stromal vascular fraction (SVF)

The SVF is derived from adipose tissue and contains a heterogeneous cell population including 10% MSCs [36, 37]. As it is not exclusively composed of MSC, we included it in this review. SVF enhances the chondrogenic capacity of chondrocytes in vitro and in mice [38]. Knowing this, Rothrauff et al. set out to use autologous SVF to augment radial tears in goats [16]. They harvested adipose tissue from the infra-patellar fat pad, prepared it to isolate SVF and then implanted it in a gelatin hydrogel. Three groups (*n* = 4 per group) of goats (1 untreated group, 1 sutured group and 1 sutured-SVF augmented group) were compared macroscopically, with magnetic resonance, and histologically 6 months after surgery. Radial tears were found to be highly arthrogenic in all cases, even when sutured. The SVF-augmented group showed more tissue formation at the level of the tear, although osteochondral degeneration was still present. This technique of harvesting fat from the infra-patellar fat pad reduces the morbidity of a distant donor site and enables a one-stage procedure, unlike autologous MSC implantation, which generally requires a two-stage surgery [39]. In addition, the numerous manipulations involved in preparing SVF increase the risk of infection, and the time between collection and reimplantation can take up to 2 h [16]. Although interesting, this technique needs to be detailed and perfected in further studies.

#### Gli1 + cells

Gli1 is recognized as a marker for bone marrow and periosteal mesenchymal progenitors [40]. In addition, meniscal injury leads to rapid division of Gli1-producing cells (Gli1 + cells) to stimulate cell migration towards the tear, although meniscal healing is not very effective [41]. Wei et al. therefore proposed the idea of injecting Gli1 + meniscal cells into a mouse after meniscal injury [41]. The concept seems interesting in vitro and focuses more specifically on meniscus-cell progenitors. However, the lack of information on in vivo testing prevents us from commenting on this cell therapy strategy.

###  Others

####  Atelocollagen

The study involving atelocollagen, a collagen derivative, for augmentation of medial meniscus root repair, is a retrospective case–control study of 47 patients, 25 of whom received this compound [15]. Here, atelocollagen was combined with fibrin glue and applied in addition to a transosseous tunnel root repair. Magnetic resonance imaging at 1-year post-op showed a lower intrameniscal signal intensity in the atelocollagen group, suggesting better healing. However, no difference in terms of meniscal extrusion or clinical score could be demonstrated, while increasing the cost for the patient. It is therefore not currently recommended to use atelocollagen to augment meniscal root repairs.

####  Simvastatin

Simvastatin is generally used to treat hyperlipidemia and prevent cardiovascular disease, but it also has other biological effects, notably on chondrocytes [42, 43]. In this context, Zhang et al. used it to stimulate the healing of cylindrical meniscus defects in avascular zones in rabbits [44]. In the study group, simvastatin was combined with a gelatin hydrogel for better local effect and release. As early as 8 weeks post-operatively, macroscopic and histological observations showed better healing in the treated group. Immunohistochemical evaluation showed that meniscal tissue in the treated group was highly positive for collagen types 1 and 2, compared with the control group. This study also had the advantage of evaluating the repair biomechanically at 12 weeks. The treated group showed stiffer tissue than the control group, although it was less resistant than healthy meniscus tissue. Simvastatin therefore is a factor of interest, as it increases local production of type 1 and 2 collagens. However, the circular defects do not reflect clinical reality, and the effect of the hydrogel alone was not evaluated in this study. Possible systemic side effects would also need to be determined.

#### Hyaluronic acid (HA)

Hyaluronic acid is usually used in the conservative treatment of osteoarthritis [37, 45]. In this systematic review, three articles studied hyaluronic acid as a meniscal augmentation agent in animal models. In Ishima et al. 20 rabbits underwent a longitudinal tear in a red-red zone left without suture treatment [46]. The study group (*n *= 10) was treated with HA injection once a week for 5 weeks, while the control group (*n* = 10) received saline. Rabbits were sacrificed at 6 and 12 weeks. HA had no beneficial effect on these longitudinal tears in the peripheral zone. In Sonoda et al. 35 white rabbits were also treated with HA injections once a week during 5 weeks (study group) or with saline injections (control group) [47]. Both groups also received Ethilon sutures. Once again, no macroscopic or histological differences were reported for tears in either the peripheral or avascular zones. Furthermore, a biomechanical study of pull-out strength showed no difference between the two groups. Suzuki et al. made cylindric defects with a needle (0.78 mm diameter) on the anterior horn of the lateral meniscus of rabbits (*n* = 24) [48]. They were treated with HA once a week or saline injections. Animals were sacrificed at 1 and 6 weeks post-operatively. At 6 weeks, the HA group showed better filling of the meniscus tear with chondrocyte-like scar tissue than the control group.

HA presents conflicting results. We hypothesize that its lubricating and anti-inflammatory effect may temporarily improve the post-traumatic inflammation of the operated knees in these studies [49]. However, the lack of evidence of any real effect and the rapid clearance of HA (not associated with a matrix) requires weekly injections, which seems unrealistic in clinical terms.

####  Double-stranded microRNA-210 (ds miRNA-210)

Ds miRNA is an RNA segment of approximately 22 nucleotides that plays an important role in gene regulation in many systems [50]. In response to hypoxia, miR-210 is a key player in angiogenesis [51]. It also appears to stimulate capillary formation and the migration of VEGF-producing cells [52]. For these characteristics, Kawanishi et al. wanted to test an intra-articular injection of ds miRNA-210 to augment longitudinal meniscal tears in avascular zones in rats [53]. At 12 weeks post-injection, the tear was filled with scar tissue in the treated group, and collagen type 2 expression was predominantly observed. Although interesting, the scar tissue formed does not have the same composition as native tissue, and the mechanism of action of this factor is not clearly understood. In addition, mi-RNA210 appears to be involved in cancer pathogenesis and is therefore not currently a factor of interest for meniscal augmentation [54, 55].

The decellularized extracellular matrix (dECM) is also the subject of numerous studies [56]. Indeed, it can support a hydrogel containing augmentation factors such as growth factors [57]. dECM can be produced from allogeneic or xenogeneic menisci [36]. It also has the advantage of having good histocompatibility and constructing a favorable differentiation microenvironment for surrounding cells [56]. Furthermore, its biomechanical properties are similar to native meniscal tissue. This type of matrix is already tested to repair cartilage with favorable results [58]. It is crucial to continue exploring this highly promising route of administration and support, which could potentially serve as the delivery platform for future optimized augmentation factors.

As previously mentioned, one of the biggest challenges in meniscal healing is the lack of vascularisation in the inner third of the meniscus, also called the white-white zone [59]. Augmentation strategies are there to facilitate meniscal healing and reduce the rate of revision surgery for suture failure. It is important to understand that late suture failure occurs because the tissue has not healed and the suture has failed due to fatigue [60].

Certainly, there are other factors besides biological augmentation that can influence the outcomes of meniscal repairs. Studies have shown that certain tear morphologies have lower success rates; for instance, radial lesions, due to their configuration, tend to reopen under axial load during walking [61]. Conversely, longitudinal vertical tears tend to be compressed under these same conditions, with suture success rates ranging between 72 and 94% [9, 62]. Additionally, surgical skills and optimal repair techniques also play a significant role. Indeed, certain tears require specific skills and expertise, such as transosseous suturing for root tears or special suture techniques for radial tears [63, 64]. Opting for meniscectomy may sometimes be simpler from a technical standpoint, which may explain why this approach is unfortunately preferred in some cases [5].

With this systematic review, we wanted to look further than the three classic augmentation strategies (PRP, MSCs and fibrin clot) to broaden our horizons and identify an emerging factor of interest. We believe that it can be helpful to take a step back from a problem and a number of interesting factors were identified, but none of them really stood out. The heterogeneity of factors, designs, study groups and the lack of biomechanical studies are among the reasons for this observation. The authors also believe that there are too many unknowns when it comes to meniscus metabolism, leading to treatment targets that are not always appropriate. For example, chondrocyte implantation will tend to produce cartilage-like tissue which is more rigid than meniscus-like tissue [19, 34, 53]. The treatment aim is to obtain scar tissue similar to healthy meniscus tissue. Also, 68% of these studies decided to incorporate the factor into a matrix. Among these, fibrin and gelatin hydrogel matrices were the most common [16, 19, 44]. This reflects awareness of rapid intra-articular clearance and the need to associate the factor with a matrix to control release kinetics over time [65]. Moreover, apart from the study by Weinand et al., no follow-up studies were carried out after publication, which may also reflect the difficulties these research teams faced when setting up this type of study [33].

Despite numerous advances and research on the subject, major challenges remain when it comes to biological augmentation for meniscus repair. One such challenge involves the release of augmentation factors, which should be regular and lasting several weeks, given the long time required for meniscal tissue healing [66, 67]. It is also crucial for the augmentation strategy to target the meniscal tear only and avoid adjacent hypertrophy or systemic effects caused by vascular diffusion. Moreover, almost all meniscal surgical interventions are performed arthroscopically, in a saline environment, which can also lead to intra-articular diffusion of the factor and decrease its local effectiveness [63]. Ideally, the augmentation factor should be cheap and stable for easy preservation and storage. Unfortunately, meniscal augmentation poses numerous challenges that can be complex to manage simultaneously, but it is what make this research topic so intriguing and important. Meniscal augmentation remains a key research avenue for the repair of this poorly understood and vascularized tissue [68, 69], letting us hope for better success rates and clinical outcomes after meniscal repair [70].

##  Conclusions

Different factors for the biological augmentation of meniscal repair have emerged, especially over the last ten years. This systematic review describes the challenges that come when trying to determine an optimal factor, although augmentation appears to be a promising strategy to improve meniscus repair outcomes. More research is needed to better understand the pathways that regulate meniscal healing and thus act in a more targeted and effective way.

## Data Availability

The datasets used and/or analysed during the current study are available from the corresponding author on reasonable request.

## Abbreviations

PRP: Platelet-rich plasma
MSCs: Mesenchymal stem cells
OA: Osteoarthritis
VEGF: Vascular endothelial growth factor
BMP-7: Bone morphogenic protein 7
CTGF: Connective tissue growth factor
FGF-2: Fibroblast growth factor 2
SVF: Stromal vascular fraction
IHC: Immunohistochemical
PCR: Polymerase chain reaction
PDLLA coated suture: poly(d,l-lactide)-coated Ethibond
HA: Hyaluronic acid
ds miRNA-210: Double-stranded microRNA-210
dECM: Decellularized extracellular matrix

## References

[1] Clayton RAE, Court-Brown CM (2008). The epidemiology of musculoskeletal tendinous and ligamentous injuries. Injury.

[2] Jacquet C, Pujol N, Pauly V, Beaufils P, Ollivier M (2019). Analysis of the trends in arthroscopic meniscectomy and meniscus repair procedures in France from 2005 to 2017. Orthop Traumatol Surg Res OTSR.

[3] Abrams GD, Frank RM, Gupta AK, Harris JD, McCormick FM, Cole BJ (2013). Trends in meniscus repair and meniscectomy in the United States, 2005–2011. Am J Sports Med.

[4] Seil R, Becker R (2016). Time for a paradigm change in meniscal repair: save the meniscus!. Knee Surg Sports Traumatol Arthrosc Off J ESSKA.

[5] Bąkowski P, Bąkowska-Żywicka K, Ciemniewska-Gorzela K, Piontek T (2022). Meniscectomy is still a frequent orthopedic procedure: a pending need for education on the meniscus treatment possibilities. Knee Surg Sports Traumatol Arthrosc.

[6] Abram SGF, Hopewell S, Monk AP, Bayliss LE, Beard DJ, Price AJ (2020). Arthroscopic partial meniscectomy for meniscal tears of the knee: a systematic review and meta-analysis. Br J Sports Med.

[7] Fairbank TJ (1948). Knee joint changes after meniscectomy. J Bone Joint Surg Br.

[8] Pengas IP, Assiotis A, Nash W, Hatcher J, Banks J, McNicholas MJ (2012). Total meniscectomy in adolescents: a 40-year follow-up. J Bone Joint Surg Br.

[9] Majewski M, Stoll R, Widmer H, Müller W, Friederich NF (2006). Midterm and long-term results after arthroscopic suture repair of isolated, longitudinal, vertical meniscal tears in stable knees. Am J Sports Med.

[10] Nepple JJ, Dunn WR, Wright RW (2012). Meniscal repair outcomes at greater than five years: a systematic literature review and meta-analysis. J Bone Joint Surg Am.

[11] Espejo-Reina A, Aguilera J, Espejo-Reina MJ, Espejo-Reina MP, Espejo-Baena A (2019). One-third of meniscal tears are repairable: an epidemiological study evaluating meniscal tear patterns in stable and unstable knees. Arthroscopy.

[12] Keller RE, O’Donnell EA, Medina GIS, Linderman SE, Cheng TTW, Sabbag OD (2022). Biological augmentation of meniscal repair: a systematic review. Knee Surg Sports Traumatol Arthrosc.

[13] Haunschild ED, Huddleston HP, Chahla J, Gilat R, Cole BJ, Yanke AB (2020). Platelet-rich plasma augmentation in meniscal repair surgery: a systematic review of comparative studies. Arthrosc J Arthrosc Relat Surg Off Publ Arthrosc Assoc N Am Int Arthrosc Assoc.

[14] Zaffagnini S, Poggi A, Reale D, Andriolo L, Flanigan DC, Filardo G (2021). Biologic augmentation reduces the failure rate of meniscal repair: a systematic review and meta-analysis. Orthop J Sports Med.

[15] Lee DW, Jang HG, Lee YJ, Moon SG, Kim NR, Kim JG (2020). Effect of atelocollagen on the healing status after medial meniscal root repair using the modified Mason-Allen stitch. Orthop Traumatol Surg Res OTSR.

[16] Rothrauff BB, Sasaki H, Kihara S, Overholt KJ, Gottardi R, Lin H (2019). Point-of-care procedure for enhancement of meniscal healing in a goat model utilizing infrapatellar fat pad-derived stromal vascular fraction cells seeded in photocrosslinkable hydrogel. Am J Sports Med.

[17] Seol D, Zhou C, Brouillette MJ, Song I, Yu Y, Choe HH (2017). Characteristics of meniscus progenitor cells migrated from injured meniscus: characteristics of meniscus progenitor cells. J Orthop Res.

[18] Hoeben A, Landuyt B, Highley MS, Wildiers H, Oosterom ATV, Bruijn EAD (2004). Vascular endothelial growth factor and angiogenesis. Pharmacol Rev.

[19] Hashimoto J, Kurosaka M, Yoshiya S, Hirohata K (1992). Meniscal repair using fibrin sealant and endothelial cell growth factor. An experimental study in dogs. Am J Sports Med..

[20] Kopf S, Birkenfeld F, Becker R, Petersen W, Stärke C, Wruck CJ (2010). Local treatment of meniscal lesions with vascular endothelial growth factor. J Bone Joint Surg Am.

[21] Petersen W, Pufe T, Stärke C, Fuchs T, Kopf S, Neumann W (2007). The effect of locally applied vascular endothelial growth factor on meniscus healing: gross and histological findings. Arch Orthop Trauma Surg.

[22] Nishida T, Nakanishi T, Asano M, Shimo T, Takigawa M (2000). Effects of CTGF/Hcs24, a hypertrophic chondrocyte-specific gene product, on the proliferation and differentiation of osteoblastic cells in vitro. J Cell Physiol.

[23] Shimo T, Nakanishi T, Nishida T, Asano M, Kanyama M, Kuboki T (1999). Connective tissue growth factor induces the proliferation, migration, and tube formation of vascular endothelial cells in vitro, and angiogenesis in vivo1. J Biochem (Tokyo).

[24] He W, Liu YJ, Wang ZG, Guo ZK, Wang MX, Wang N (2011). Enhancement of meniscal repair in the avascular zone using connective tissue growth factor in a rabbit model. Chin Med J (Engl).

[25] Rifkin DB, Moscatelli D (1989). Recent developments in the cell biology of basic fibroblast growth factor. J Cell Biol.

[26] Narita A, Takahara M, Sato D, Ogino T, Fukushima S, Kimura Y (2012). Biodegradable gelatin hydrogels incorporating fibroblast growth factor 2 promote healing of horizontal tears in rabbit meniscus. Arthrosc J Arthrosc Relat Surg Off Publ Arthrosc Assoc N Am Int Arthrosc Assoc.

[27] Sandler AB, Scanaliato JP, Raiciulescu S, Nesti L, Dunn JC (2023). Bone morphogenic protein for upper extremity fractures: a systematic review. HAND.

[28] Forriol F, Ripalda P, Duart J, Esparza R, Gortazar AR (2014). Meniscal repair possibilities using bone morphogenetic protein-7. Injury.

[29] Zellner J, Taeger CD, Schaffer M, Roldan JC, Loibl M, Mueller MB (2014). Are applied growth factors able to mimic the positive effects of mesenchymal stem cells on the regeneration of meniscus in the avascular zone?. BioMed Res Int.

[30] King TV, Vallee BL (1991). Neovascularisation of the meniscus with angiogenin. An experimental study in rabbits. J Bone Joint Surg Br..

[31] Gao X, Cheng Q, Zhang X, Zhao G (2020). Successful total hip arthroplasty for autosomal dominant osteopetrosis complicated by hip osteoarthritis: A case report and review of the literature. Exp Ther Med.

[32] Peretti GM, Gill TJ, Xu JW, Randolph MA, Morse KR, Zaleske DJ (2004). Cell-based therapy for meniscal repair: a large animal study. Am J Sports Med.

[33] Weinand C, Peretti GM, Adams SB, Randolph MA, Savvidis E, Gill TJ (2006). Healing potential of transplanted allogeneic chondrocytes of three different sources in lesions of the avascular zone of the meniscus: a pilot study. Arch Orthop Trauma Surg.

[34] Weinand C, Peretti GM, Adams SB, Bonassar LJ, Randolph MA, Gill TJ (2006). An allogenic cell-based implant for meniscal lesions. Am J Sports Med.

[35] Salzmann GM, Ossendorff R, Gilat R, Cole BJ (2021). Autologous minced cartilage implantation for treatment of chondral and osteochondral lesions in the knee joint: an overview. Cartilage.

[36] Gentile P, Sterodimas A, Pizzicannella J, Dionisi L, De Fazio D, Calabrese C (2020). Systematic review: allogenic use of Stromal Vascular Fraction (SVF) and decellularized Extracellular Matrices (ECM) as Advanced Therapy Medicinal Products (ATMP) in tissue regeneration. Int J Mol Sci.

[37] Belk JW, Kraeutler MJ, Houck DA, Goodrich JA, Dragoo JL, McCarty EC (2021). Platelet-rich plasma versus hyaluronic acid for knee osteoarthritis: a systematic review and meta-analysis of randomized controlled trials. Am J Sports Med.

[38] Wu L, Prins HJ, Leijten J, Helder MN, Evseenko D, Moroni L (2016). Chondrocytes cocultured with stromal vascular fraction of adipose tissue present more intense chondrogenic characteristics than with adipose stem cells. Tissue Eng Part A.

[39] Korpershoek JV, de Windt TS, Hagmeijer MH, Vonk LA, Saris DBF (2017). Cell-based meniscus repair and regeneration: at the brink of clinical translation?: a systematic review of preclinical studies. Orthop J Sports Med.

[40] Makris EA, Hadidi P, Athanasiou KA (2011). The knee meniscus: structure-function, pathophysiology, current repair techniques, and prospects for regeneration. Biomaterials.

[41] Wei Y, Sun H, Gui T, Yao L, Zhong L, Yu W, et al. Identification of Gli1 as a progenitor cell marker for meniscus development and injury repair. bioRxiv. 2020;(nov)2020–11. 10.1101/2020.11.27.401463.

[42] Gilbertson L, Ahn SH, Teng PN, Studer RK, Niyibizi C, Kang JD (2008). The effects of recombinant human bone morphogenetic protein-2, recombinant human bone morphogenetic protein-12, and adenoviral bone morphogenetic protein-12 on matrix synthesis in human annulus fibrosis and nucleus pulposus cells. Spine J.

[43] Hu M, Hung L, Yang S, Sun Y, Shih TT, Lin F (2011). Lovastatin promotes redifferentiation of human nucleus pulposus cells during expansion in monolayer culture. Artif Organs.

[44] Zhang S, Matsushita T, Kuroda R, Nishida K, Matsuzaki T, Matsumoto T (2016). Local administration of simvastatin stimulates healing of an avascular meniscus in a rabbit model of a meniscal defect. Am J Sports Med.

[45] Costa FR, Costa Marques MR, Costa VC, Santos GS, Martins RA, Santos MD (2023). Intra-articular hyaluronic acid in osteoarthritis and tendinopathies: molecular and clinical approaches. Biomedicines.

[46] Ishima M, Wada Y, Sonoda M, Harada Y, Katsumi A, Moriya H (2000). Effects of hyaluronan on the healing of rabbit meniscus injured in the peripheral region. J Orthop Sci Off J Jpn Orthop Assoc.

[47] Sonoda M, Harwood FL, Amiel ME, Moriya H, Temple M, Chang DG (2000). The effects of hyaluronan on tissue healing after meniscus injury and repair in a rabbit model. Am J Sports Med.

[48] Suzuki Y, Takeuchi N, Sagehashi Y, Yamaguchi T, Itoh H, Iwata H (1998). Effects of hyaluronic acid on meniscal injury in rabbits. Arch Orthop Trauma Surg.

[49] Jin L, Xu K, Liang Y, Du P, Wan S, Jiang C (2022). Effect of hyaluronic acid on cytokines and immune cells change in patients of knee osteoarthritis. BMC Musculoskelet Disord.

[50] Bartel DP (2004). MicroRNAs: genomics, biogenesis, mechanism, and function. Cell.

[51] Fasanaro P, Greco S, Lorenzi M, Pescatori M, Brioschi M, Kulshreshtha R (2009). An integrated approach for experimental target identification of hypoxia-induced miR-210 *. J Biol Chem.

[52] Fasanaro P, D’Alessandra Y, Stefano VD, Melchionna R, Romani S, Pompilio G (2008). MicroRNA-210 modulates endothelial cell response to hypoxia and inhibits the receptor tyrosine kinase ligand Ephrin-A3 *. J Biol Chem.

[53] Kawanishi Y, Nakasa T, Shoji T, Hamanishi M, Shimizu R, Kamei N (2014). Intra-articular injection of synthetic microRNA-210 accelerates avascular meniscal healing in rat medial meniscal injured model. Arthritis Res Ther.

[54] Sabry D, El-Deek SEM, Maher M, El-Baz MAH, El-Bader HM, Amer E (2019). Role of miRNA-210, miRNA-21 and miRNA-126 as diagnostic biomarkers in colorectal carcinoma: impact of HIF-1α-VEGF signaling pathway. Mol Cell Biochem.

[55] Li X, Yuan M, Song L, Wang Y (2020). Silencing of microRNA-210 inhibits the progression of liver cancer and hepatitis B virus-associated liver cancer via targeting EGR3. BMC Med Genet.

[56] Monibi FA, Cook JL (2017). Tissue-derived extracellular matrix bioscaffolds: emerging applications in cartilage and meniscus repair. Tissue Eng Part B Rev.

[57] Li Z, Yan W, Zhao F, Wang H, Cheng J, Duan X (2023). Regional specific tunable meniscus decellularized extracellular matrix (MdECM) reinforced bioink promotes anistropic meniscus regeneration. Chem Eng J.

[58] Das P, Singh YP, Mandal BB, Nandi SK. Chapter 10 - Tissue-derived decellularized extracellular matrices toward cartilage repair and regeneration. In: Caballero D, Kundu SC, Reis RL, editors. Methods in Cell Biology. Academic Press; p. 185–221. (Cell-derived Matrices - Part B; vol. 157). 2020. 10.1016/bs.mcb.2019.11.005 32334715

[59] Rubman MH, Noyes FR, Barber-Westin SD (1998). Arthroscopic repair of meniscal tears that extend into the avascular zone. A review of 198 single and complex tears. Am J Sports Med..

[60] Ronnblad E, Barenius B, Engstrom B, Eriksson K (2020). Predictive factors for failure of meniscal repair: a retrospective dual-center analysis of 918 consecutive cases. Orthop J Sports Med.

[61] Greis PE, Bardana DD, Holmstrom MC, Burks RT (2002). Meniscal injury: I. Basic science and evaluation. JAAOS - J Am Acad Orthop Surg.

[62] Beaufils P, Pujol N (2017). Management of traumatic meniscal tear and degenerative meniscal lesions. Save the meniscus. Orthop Traumatol Surg Res.

[63] Beaufils P, Pujol N (2018). Meniscal repair: Technique. Orthop Traumatol Surg Res.

[64] Bhatia S, Civitarese DM, Turnbull TL, LaPrade CM, Nitri M, Wijdicks CA (2016). A novel repair method for radial tears of the medial meniscus: biomechanical comparison of transtibial 2-tunnel and double horizontal mattress suture techniques under cyclic loading. Am J Sports Med.

[65] Freedman BR, Uzun O, Luna NMM, Rock A, Clifford C, Stoler E (2021). Degradable and removable tough adhesive hydrogels. Adv Mater.

[66] Li J, Mooney DJ (2016). Designing hydrogels for controlled drug delivery. Nat Rev Mater.

[67] de Albornoz PM, Forriol F (2012). The meniscal healing process. Muscles Ligaments Tendons J.

[68] Henning CE, Lynch MA, Clark JR (1987). Vascularity for healing of meniscus repairs. Arthrosc J Arthrosc Relat Surg.

[69] van Schie P, van der Lelij TJN, Gerritsen M, Meijer RPJ, van Arkel ERA, Fiocco M (2022). Intra-operative assessment of the vascularisation of a cross section of the meniscus using near-infrared fluorescence imaging. Knee Surg Sports Traumatol Arthrosc.

[70] DePhillipo NN, LaPrade RF, Zaffagnini S, Mouton C, Seil R, Beaufils P (2021). The future of meniscus science: international expert consensus. J Exp Orthop.

